# Positive response of a recurrent clear cell sarcoma to anlotinib combined with chemotherapy: A case report

**DOI:** 10.1097/MD.0000000000032109

**Published:** 2022-12-02

**Authors:** Junyue Tao, Hao Yang, Zongyao Hao, Chaozhao Liang, Yingying Du, Chao Zhang, Yu Yin, Jun Zhou

**Affiliations:** a Department of Urology, The First Affiliated Hospital of Anhui Medical University, Hefei, Anhui, People’s Republic of China; b Department of Oncology, The First Affiliated Hospital of Anhui Medical University, Hefei, Anhui, People’s Republic of China; c Department of Hepatobiliary Surgery, The First Affiliated Hospital of Anhui Medical University, Hefei, Anhui, People’s Republic of China; d Department of Pathology, The First Affiliated Hospital of Anhui Medical University, Hefei, Anhui, People’s Republic of China.

**Keywords:** anlotinib, chemotherapy, neoadjuvant, recurrent clear cell sarcoma

## Abstract

Renal clear cell sarcoma is a rare and highly invasive malignant renal tumor that easily relapses after treatment. Recurrent recurrent clear cell carcinoma (CCSK) responds poorly to chemotherapy and has no established standardized treatment, and need to be explored potentially useful treatments.

**Patient concerns:**

A 18-years-old patient with renal clear cell sarcoma recurrence after open radical nephrectomy.

**Diagnosis:**

Recurrent clear cell sarcoma.

**Interventions:**

After chemotherapy alone failed, the patient received 6 courses of anlotinib combined with chemotherapy. The tumor had significantly reduced in size and the recurrent tumor and part of the liver were resected.

**Outcomes:**

No tumor recurrence or metastasis was detected during the follow-up 8 months after the operation.

**Lessons:**

This is the first report describing the use of anlotinib in treating CCSK. We believe that anlotinib combined with chemotherapy may be a useful treatment option for patients with recurrent CCSK.

## 1. Introduction

Renal clear cell sarcoma (CCSK) is a rare malignant renal tumor in children, and the current age-adjusted incidence of childhood CCSK is 0.205 per million.^[1]^ At present, in addition to surgical treatment, traditional systemic chemotherapy, that is, the alternating use of cyclophosphamide, etoposide, carboplatin, vincristine, doxorubicin, and other chemotherapeutic drugs, is also utilized. Through the current treatment regimen, the results of CCSK were significantly improved [5-years event-free survival (EFS) and overall survival (OS) rates were 79% and 90%, respectively].^[2]^ Although the survival rate has improved, the recurrence rate is still high, especially among young patients and those with advanced disease, with about 16% of patients experiencing recurrence.^[3–5]^ Recurrent CCSK is often insensitive to chemotherapy and has a very poor prognosis.^[6]^ Therefore, it is necessary to explore new treatment strategies to maximize the survival rates of these patients.

Anlotinib is a new, orally administered tyrosine kinase inhibitor (TKI) that targets platelet-derived growth factor receptors (PDGFR), c-KIT, vascular endothelial growth factor receptor (VEGFR), and fibroblast growth factor receptor (FGFR).^[7]^ Anlotinib has been found to offer satisfactory results in the treatment of a variety of sarcomas.^[8–10]^ However, its applicability for the treatment of CCSK has not been reported. Here, we describe the first case of recurrent CCSK that responded well to a combination of anlotinib and chemotherapy, after chemotherapy alone failed.

## 2. Case presentation

**Figure 1.**
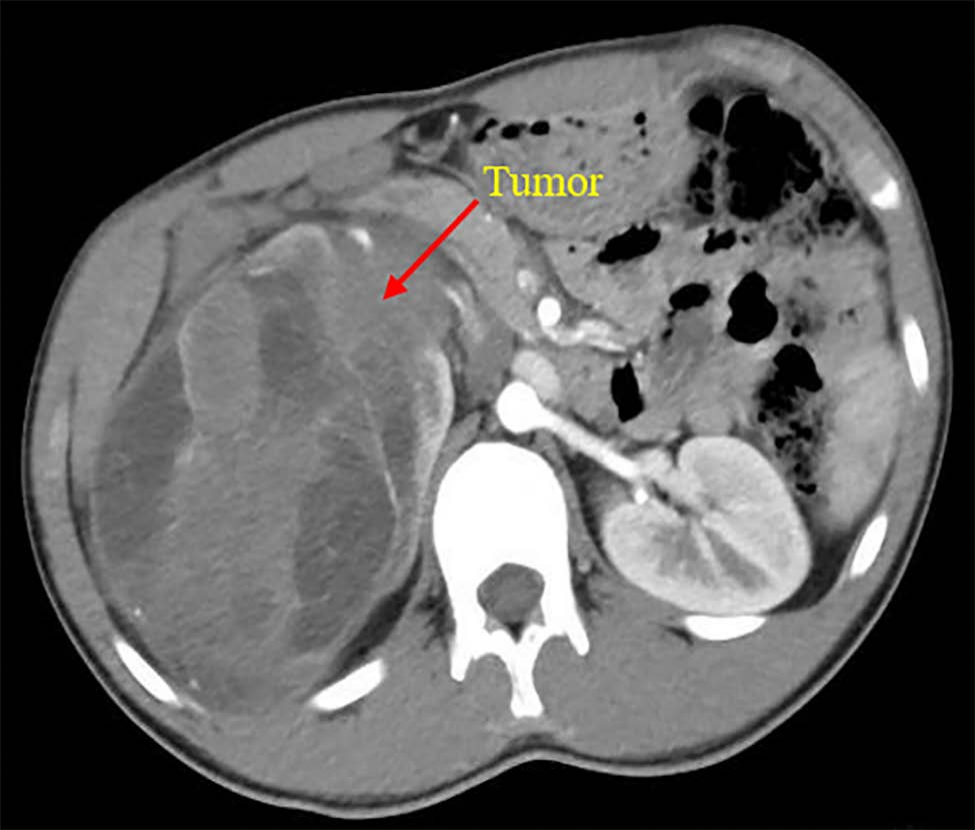
The first preoperative MRI of the patient shows a large tumor of the right kidney. MRI = magnetic resonance imaging.

**Figure 2.**
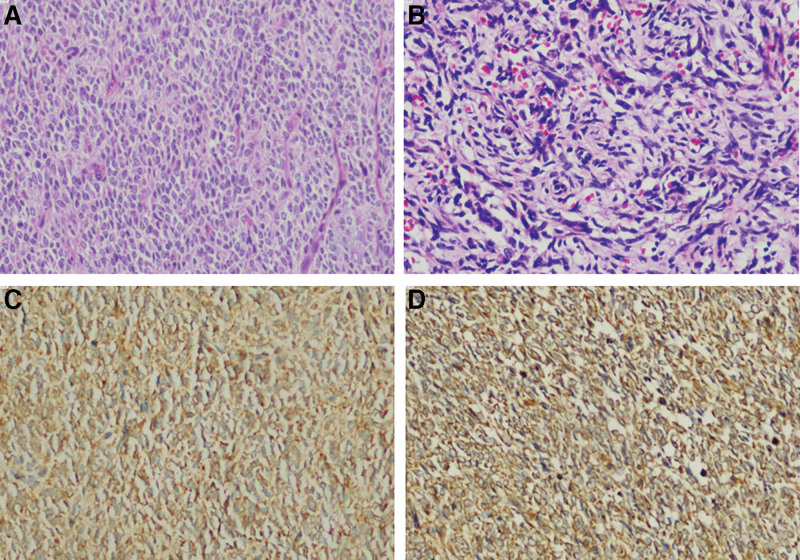
The microscopic examination of the biopsy specimen (A: primary tumor, B: recurrent tumor) demonstrates a myxoid tumor with spindle- to oval-shaped cells with euchromatic nuclei and clear cytoplasm with arcuate vasculature. The tumor cells were immunohistochemically positive for Vimentin (C) and Bcl-2 (D).

An 18-years-old boy presented to the First Affiliated Hospital of Anhui Medical University, Hefei, China, for an examination following a complaint of pain on the right side of the waist. On January 21, 2020, a large tumor in the right kidney (size: 13 × 11 × 10 cm) was detected on magnetic resonance imaging (MRI), and the mass was supplied by branches of the right renal artery and surrounded by enlarged lymph nodes (Fig. 1). He underwent open radical nephrectomy on January 26; the scope of resection included the right kidney, perirenal fat, adrenal gland, upper ureter, and retroperitoneal lymph nodes. CCSK was confirmed on postoperative pathology. Immunohistochemical staining showed that the tumor cells were positive for vimentin and Bcl-2 but negative for *S*-100, desmin Syn, WT-1, EMA, CD34, and CD99; the Ki-67 index in tumor cells was 40% (Fig. 2). After recovery and discharge, the patient did not undergo postoperative chemotherapy or follow-up for personal reasons.

**Figure 3.**
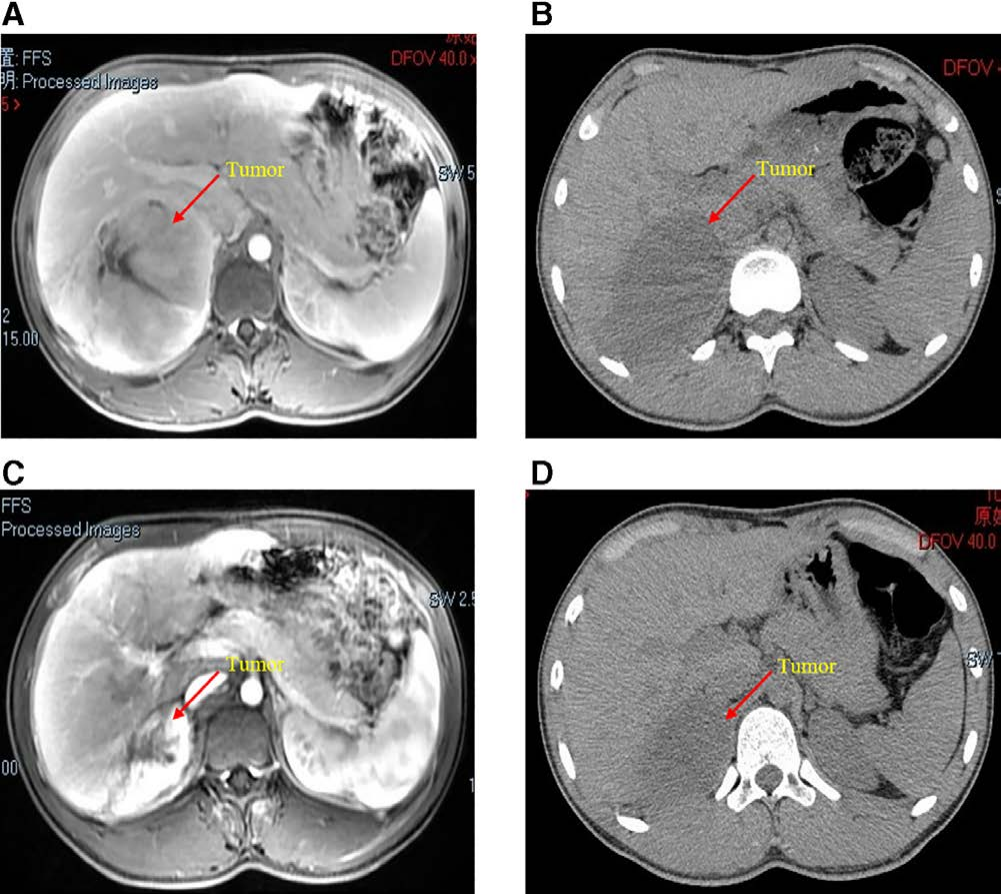
The MRI (A) of the patient shows a recurrent mass in the operative area, which adhered closely to the right lobe of the liver. The CT scans show that the mass gradually shrinks after the second (B), fourth (C), and sixth cycles (D) of neoadjuvant therapy. CT = computed tomography, MRI = magnetic resonance imaging.

**Figure 4.**
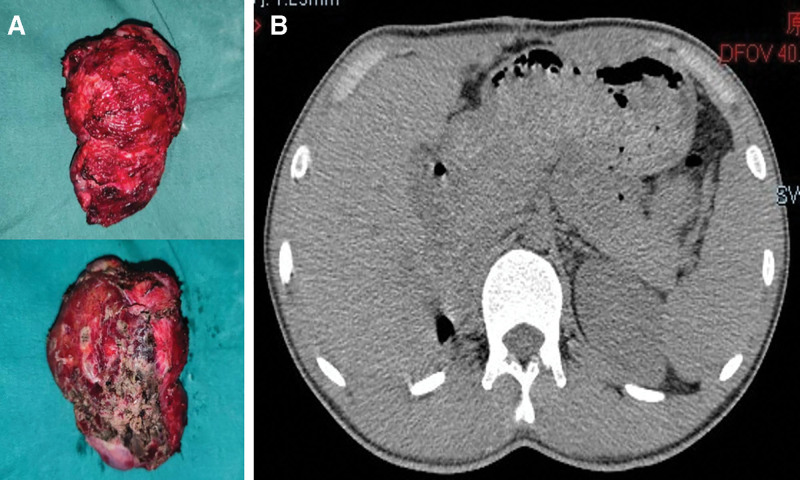
The specimens of surgical resection after neoadjuvant therapy in A, and postoperative CT presented no tumor residue at 3 months in B. CT = computed tomography.

Unfortunately, 13 months later, he again visited the hospital with a complaint of backache, and MRI revealed another mass in the operative area (size: 12 × 11 × 8 cm) on March 14, 2021 (Fig. 3A). Because the lesion had adhered to the right lobe of the liver, direct surgical resection was extremely difficult. First, we decided to treat the patient with neoadjuvant chemotherapy [doxorubicin (40 mg intravenous infusion for 1 day) plus ifosfamide (2000 mg intravenous infusion for 1/2 weeks)] to reduce the tumor volume, and then remove the tumor completely. Unfortunately, the patient was completely unresponsive to chemotherapy, and there was no significant change in tumor size after 3 courses. After a multidisciplinary team meeting involving the urology, hepatobiliary surgery, and oncology departments, we decided to treat the patient with anlotinib combined with chemotherapy. Anlotinib (12 mg oral 1/day for 14 consecutive days) plus doxorubicin (40 mg intravenous infusion for 1 day) plus ifosfamide (2000 mg intravenous infusion for 1/2 weeks) for 6 cycles of treatment were administered beginning July 2, 2021. During this period, the patient was treated with adjuvant therapy to prevent adverse events such as infection and nausea, and no serious adverse reactions occurred. The tumor shrank gradually over the course of treatment (Fig. 3B and C), and on January 7, 2022, MRI showed that the tumor size was significantly reduced (approximately 6 × 6 × 4 cm in size) (Fig. 3D). Resection of the right retroperitoneal tumor (including the right tumor and part of the right lobe of the liver) was performed on January 21, 2022 (Fig. 4A). Three months after the operation, computed tomography showed that there was no residual tumor in the surgical area (Fig. 4B). To prevent another relapse, Anlotinib combined with chemotherapy was continued for 6 cycles after surgery, and the patient is currently being followed up.

## 3. Discussion

Since Morgan reported the first case of CCSK, whose histological features are completely different from those of Wilms’ tumor (WT) in 1970,^[11]^ diagnosing and treating this condition has been a challenge because of its rarity. Moreover, the clinical manifestations of CCSK are not specific and include abdominal distension or mass, low back pain, and gross hematuria, making it difficult to distinguish from other renal tumors, especially WT.^[12]^ At the first visit, our patient showed a huge mass in the right abdomen with low back pain and particularly rich blood vessels were found inside the tumor on computed tomography and MR examinations. First, our diagnostic hypothesis was WT, which is the most common renal tumor in children.^[13]^ According to the 2001 European Agreement of the International Society of Pediatric Oncology, percutaneous biopsy of renal tumors is not systematically recommended.^[14]^ Therefore, renal biopsy was not performed before surgery, and CCSK was confirmed by pathology after the first operation. It has been reported that immunohistochemical analysis is helpful to distinguish CCSK from other renal tumors in children because CCSK is often positive for Vimentin and Bcl-2 but negative for *S*-100, desmin Syn, WT-1, EMA, CD34, and CD99.^[15]^ The immunohistochemical results for this patient were consistent with these results. Unfortunately, the patient declined the treatment we offered after the first surgery, and the tumor recurred 16 months later.

As a rare and highly invasive renal malignant tumor, CCSK is usually noted as having “‘unfavorable histology’” in the National Wilms’ Tumor Research Group (NWTS), and the survival rate of patients with CCSK is even lower than those with WT.^[16,17]^ Current international treatment guidelines include the European UMBRELLA International Society of Pediatric Oncology (SIOP) RTSG 2016 protocol and the North American NWTS-Children’s Oncology Group guidelines.^[18,19]^ The SIOP protocol advocates preoperative chemotherapy, whereas the Children’s Oncology Group guidelines advocate surgical resection first. There was no significant difference in survival rates between the 2 treatment regimens, and the treatment recommendations depended on the stage of the tumor. For example, in the current SIOP protocol, preoperative chemotherapy with actinomycin and vincristine is indicated for stages I to III, with the addition of doxorubicin for stage IV disease. Following the initial surgical resection, postoperative chemotherapy comprises etoposide, carboplatin, and ifosfamide, alternating with cyclophosphamide and doxorubicin. Abdominal radiotherapy was administered for stage II to IV disease. However, even with these enhanced treatments, CCSK still has a considerable recurrence rate of approximately 16%.^[20]^ Unfortunately, there is no standard treatment for CSK recurrence. Gooskens conducted a statistical study on the prognosis of 37 patients with recurrent CCSK who were admitted to the SIOP and the Italian Society of Pediatric Oncology (AIEOP) from 1992 to 2012.^[21]^ The results showed that even after 30 patients received more than the normal dose of chemotherapy, the 5-years survival rate was only 26%. More importantly, a study found that if the recurrent tumor originated from the microclone of the previous tumor, the size of the recurrent tumor did not shrink after radiotherapy and chemotherapy but still existed and progressed rapidly.^[22]^ This suggests that the effect of traditional chemotherapy on recurrent CCSK is relatively poor, especially in our case, in which the recurring tumor was strongly suspected to have originated from the microclones of previous tumors and, likewise, was completely unresponsive to chemotherapy alone.

In our case, the low responsiveness of traditional chemotherapy regimens prompted us to find better treatments. Previous research has identified potential therapeutic targets on the basis of their up-regulation in CCSK, including EGFR, KIT, and PDGFRα.^[23,24]^ TKIs may become our treatment of choice, because they can significantly inhibit tumor proliferation, vasculature, and the tumor microenvironment by selectively targeting these receptor molecules.^[25]^ For example, the literature reported that TKIs can significantly delay disease progression and prolong survival in the treatment of EGFR-positive non-small cell lung cancer patients.^[26]^ Likewise, patients with gastrointestinal stromal tumor caused by activating c-KIT or PDGFR have also responded well to TKIs.^[27]^ More importantly, clinical studies have provided strong evidence that the use of anti-angiogenic agents, and particularly anlotinib, can promote tumor vascular normalization, thereby improving the delivery and efficacy of chemotherapy drugs in many solid tumors.^[28–30]^ In our case, 6 courses of treatment with anlotinib combined with chemotherapy significantly reduced the tumor size, providing an opportunity for surgical resection of the tumor, suggesting that anlotinib combined with chemotherapy may be useful in the treatment of recurrent CCSK.

To our knowledge, this is the first report on the efficacy of anlotinib combined with chemotherapy in the treatment of recurrent CCSK. Through our exploration, we believe that anlotinib combined with chemotherapy may be a useful strategy for unresectable recurrent CCSK; however, the extent to which this approach can inhibit tumors requires further research. Future research should explore the specific molecular mechanism of anlotinib for recurrent CCSK and whether combination therapy is truly useful for recurrent CCSK.

## Author contributions

**Conceptualization:** Jun Zhou.

**Formal analysis:** Jun Zhou.

**Funding acquisition:** Jun Zhou.

**Investigation:** Chaozhao Liang.

**Methodology:** Yingying Du, Chao Zhang.

**Resources:** Chao Zhang, Yu Yin.

**Software:** Chaozhao Liang.

**Supervision:** Zongyao Hao, Jun Zhou.

**Writing – original draft:** Junyue Tao, Hao Yang.

**Writing – review & editing:** Junyue Tao, Hao Yang.
